# Patterns of the Consumption of Young Children Formula in Chinese Children Aged 1–3 Years and Implications for Nutrient Intake

**DOI:** 10.3390/nu12061672

**Published:** 2020-06-04

**Authors:** Jian Zhang, Dantong Wang, Yumei Zhang

**Affiliations:** 1 School of Public Health, Peking University, Beijing 100191, China; zhangjian92@pku.edu.cn; 2 Nestle Research, CH-1000 Lausanne, Switzerland; dantong.wang@rdls.nestle.com

**Keywords:** nutrient intake, young children formula, milk, dietary modelling

## Abstract

The consumption of young children formula (YCF) is associated with reduced risk of inadequacies of nutrients that are frequently lacking in the diets of young children. In this study, we assessed the role of YCF in children’s diets and whether meeting dairy intake recommendations would improve nutrient intake in young Chinese children aged 12–36 months. Dietary intake data for children from the 2012 China Maternal and Infant Nutrition and Growth study were analyzed (n = 910). Nutrient intake was compared between YCF consumers and non-consumers, and the theoretical impact of meeting dairy intake recommendations by adding cow’s milk or YCF to children’s diets was assessed using diet modelling. The percent of children consuming YCF was 64.5% and was positively associated with family income and mother’s education level. Compared to non-consumers, YCF consumers had higher intakes of minerals (e.g., calcium, iron) and vitamins (e.g., C, D, B6) that are important for growth and immune function, and lower intakes of saturated fat. To meet dairy intake recommendations by adding either cow’s milk or YCF to children’s diets would improve intakes of vitamins and minerals in young Chinese children. YCF consumption contributes to the improvement of nutrient intakes in children aged 12–36 months in China.

## 1. Introduction

Young children formulas (YCF), also called follow-up formulas (FUF) [1] or growing up milk (GUM) [2], are milk-based formulas intended to partially satisfy the nutritional requirements of young children [3,4]. The nutritional role of YCF in children’s total diet has been studied. We reported previously that 50%–70% of children aged 1–3 years consumed YCF in China [5] and it provided 11%–22% of their total daily energy [6]. Li et al. also found that YCF was a major contributor to energy and nutrient intakes in 7- to 24-month-old children in China [7]. Vandenplasall and colleagues reviewed publications between 1990 and 2014, and concluded that in all publications reviewed, YCF helped to cover nutritional requirements of 12- to 36-month-old children [8].

Cow’s milk is another commonly consumed food in toddlers in some countries, including Mexico and the US. It has been reported that in Mexico, more than 86% of children aged 1–3 years consumed cow’s milk on any given day [9], providing 10%–13% of daily energy [10]. However, in China, cow’s milk provided only 4%–5% of daily energy intake due to a low percent of consumption, especially among children aged 1–2 years [5,6].

Several studies have been conducted to compare nutrient intakes of children consuming YCF and cow’s milk. In a French study in 1–2-year-old children, YCF consumers had significantly reduced risk of insufficiency of iron, vitamin C, and vitamin D compared to those consuming cow’s milk [11]. A recent clinical trial in Australia also showed that the consumption of YCF was associated with higher nutritional adequacy and an increased likelihood of meeting nutrient requirements in children aged 1–2 years compared to the unfortified cow’s milk group [12]. Diet modelling approaches have also been used to examine the theoretical impact of YCF on the nutrient intake of young children. In the UK, Eussen and colleagues showed that replacement of cow’s milk intake by an equal volume of YCF in young children (1–1.5 years of age) improved vitamin D and iron adequacy [13]. Similar findings were also reported by Walton and Flynn in Irish children aged 1–2 years [14].

From 2008 to 2013, the prevalence of YCF consumption among toddlers aged 1–3 years in China has increased rapidly [15]. Assessing the role of YCF in the total diet of Chinese children and comparing nutrient intakes of YCF and cow’s milk consumers is needed to understand if these beverages offer any nutritional benefits. So far, information on this topic is very limited in China. Thus, in the present study, we assessed the nutritional role of YCF by comparing the nutrient intakes between YCF consumers and non-consumers. Furthermore, we hypothesized that adding either cow’s milk or YCF to children’s diets would improve nutrient intakes in young Chinese children. To achieve this, we applied a diet modelling approach to assess the impact of increasing consumption of YCF and cow’s milk to meet dairy recommendations in Chinese children aged 12–36 months. This approach would help to improve the nutritional status of young children.

## 2. Materials and Methods

### 2.1. Study Population

Data used in the present analysis are from the Maternal and Infant Nutrition and Growth (MING) study, a cross-sectional survey conducted in 2011–2012. In MING study, dietary intake information was collected for infants and toddlers aged 6–36 months in 8 Chinese cities. Details of the study design and methodology were described previously [5]. Briefly, 2 maternal and childcare centers (MCCC) were selected in each city and 2 hospitals were selected in one city (i.e., one in the city center and another in a suburban area). MCCCs are the primary health care facilities that provide birth-related health services in China. Research protocols were approved by the Medical Ethics Research Board of Peking University (No. IRB00001052-11042) and written consent forms were obtained from the children’s primary caregivers. In total, 910 children aged 12 to 36 months were included in this study.

### 2.2. Measurement of Dietary Intakes

Dietary intakes of toddlers aged 12–36 months were collected using one 24 h recall. The recalls were conducted by trained interviewers with the caregiver who was primarily responsible for feeding the child. The caregiver was asked to provide detail about all foods, beverages, and dietary supplements consumed by the child during the day prior to the interview. Information about ingredients in homemade foods and foods consumed outside the home was also recorded. A booklet with pictures of commonly consumed foods, along with measuring aids such as spoons, cups, and bowls, was used to estimate quantities consumed. Completed recalls were coded using a purpose-built food composition database based on the Chinese Food Composition tables from 2004 and 2009 [16,17]. Since there were only a limited number of food items with values for vitamins B6 and B12 and no vitamin D value in the Chinese food composition tables, a group of experts at Peking University imputed these nutrients from similar foods found in the Japanese food composition tables [18]. In total, the intakes of 23 nutrients were included in the analysis (carbohydrates, protein, fat, saturated fat, dietary fiber, vitamin A, thiamin, riboflavin, niacin, vitamin B6, folate, vitamin B12, vitamin C, vitamin D, vitamin E, calcium, iron, zinc, selenium, sodium, and potassium).

### 2.3. Young Children Formula Consumers

In this study, fortified milk-based products intended for consumption by children aged 1–3 years, such as stage 3 formula, were classified as YCFs. YCF nutrient compositions were assessed based on data in 2004 Chinese Food Composition database [17], complemented by nutrition labels on the product packages. YCF consumers were children who reported YCF consumption in the questionnaires. Both YCF consumers and non-consumers might consume cow’s milk.

### 2.4. Measurement of Sociodemographic Characteristics

The demographic information was collected as part of the general questionnaire. Family income was classified as low, middle, or high, corresponding to families with a household monthly income per person below 2000 RMB, 2000–4000 RMB, and over 4000 RBM, respectively. Maternal education level was classified based on the years of completed schooling. Low, middle, and high education levels were defined as less than 9 years, 9–12 years, and above 12 years of schooling, respectively.

### 2.5. Statistical Methods and Modelling Scenarios

Chi-square tests were used to analyze the relationships between the percent of children consuming YCF and age group, sex, family income, and maternal education level. Due to the abnormality of intake distribution, Wilcoxon signed-rank tests were used to compare the energy and nutrient intakes between YCF consumer and non-consumer groups, while Bonferroni correction was applied for multiple comparisons. Multivariate regression models were used to assess YCF consumption and nutrient intake, adjusted by age, sex, energy intake, family income, and maternal education level. Nutrient intakes were log-transformed and values were treated as dependent variables in the regression. R version 3.6.1 was used in the estimation of the nutrient intakes and comparison analyses.

We also modelled the addition of cow’s milk or YCF for children who did not meet the daily dairy product intake recommendation of 300 g/d [19]. An amount of cow’s milk or YCF was added so that each child met the recommendation and then the nutrient intakes before addition (baseline), after adding cow’s milk (model 1), and after adding YCF (model 2) were compared. In the diet modelling, the nutrition composition of a YCF from Wyeth Nutrition was used to model the nutrient intake.

## 3. Results

### 3.1. Characteristics of Study Population and Their YCF Consumption

The proportions of children consuming YCF by demographic characteristics are shown in Table 1. The prevalence of children consuming YCF was more than 70% in younger (12–23 months) and 55% in older (24–36 months) age groups. No difference was observed between boys and girls. The prevalence of YCF consumption increased along with the family income level in young children (12–23 months). Mother’s education level was also positively associated with the percentages of children consuming YCF in both younger and older children. It is likely that there was an interaction between family income and mother’s education levels. Since this was not the focus of this study, no further stratification was performed.

### 3.2. Energy and Nutrient Intakes of YCF Consumers and Non-Consumers

To assess the nutritional benefit of YCF consumption in the total diet, children aged 12–36 months were divided into 2 groups based on whether they consumed YCF (YCF consumers and non-consumers), then energy and nutrient intakes were assessed. In total, there were 583 consumers and 327 non-consumers. As shown in Table 2, the energy intakes were around 970 kcal in both groups, which is in line with their respective estimated energy requirements (EER). No statistically significant differences were found between the two groups. Compared to non-consumers, the YCF consumer group had similar intakes of protein, total fat, carbohydrates, and dietary fiber, but lower intakes of saturated fat. YCF consumers had higher intakes of most vitamins and minerals, including vitamin A, thiamin, riboflavin, vitamin B6, folic acid, vitamins B12, vitamin C, vitamin D, vitamin E, calcium, iron, zinc, and potassium. The only micronutrient that was lower in the YCF group as compared to non-consumers was magnesium.

### 3.3. Modelling Nutrient Intakes to Meet the Dairy Intake Recommendations

In the present study, 424 children did not meet the dairy intake recommendation from the Chinese Nutrition Society (i.e., 300 g/d) [19]. To evaluate the nutritional impact of meeting the recommendation, we modelled the impact of two approaches—supplementing the children’s diet with cow’s milk (model 1) or a YCF (model 2) so that the total dairy intake for each child reached 300 g/d. We then compared the nutrient intakes of the two scenarios with reported consumption (baseline). On average, 122 ± 71 g (mean ± SD) of milk or YCF was added. The nutrient intakes of each scenario are shown in Table 3. Unsurprisingly, the energy intakes were higher in the two addition models; the median energy intake increased from 867 kcal (reported) to 945 kcal (model 1) and 968 kcal (model 2). Model 1, involving addition of cow’s milk, was the highest in protein (34.0 g/d), total fats (32.3 g/d), and saturated fats (8.3 g/d). Cow’s milk addition also resulted in the highest intakes of riboflavin, calcium, phosphorus, magnesium, and sodium. On the other hand, model 2, involving addition of YCF, was the highest in carbohydrates, dietary fiber, vitamin A, thiamin, niacin, vitamin B6, folate, vitamin B12, vitamin C, vitamin D, iron, zinc, selenium, and potassium.

## 4. Discussion

YCF is commonly consumed among Chinese toddlers. In the present study, we found that 71% and 56% of children consumed YCF at 12–23 months and 24–35 months of age, respectively. These results were consistent with a previous report from China [5] but higher than results from the Philippines (22% and 8% for 1–2 years and 3–4 years, respectively) [20]. In this study, we further analyzed the association of family income and maternal education levels with YCF consumption and found that higher-income families and mothers with higher education levels were more likely to provide YCF to their children, similar to results found in the Philippines [20].

In this study, we concluded that YCF consumers generally had better nutrient intake than non-consumers based on the following three findings. First, there was no difference in energy intake between YCF consumers and non-consumers, as the mean intakes for both groups were in line with their EER for each age group (i.e., 800–1000 kcal). Second, although the total fat intakes were similar, the saturated fat intake was lower in YCF consumers, suggesting a better fatty acid intake profile. Third, many micronutrient intakes (such as iron, calcium vitamin C, vitamin D, vitamin E, vitamin B6, and folate, which are important for growth and immune function) were higher in YCF consumers, highlighting the nutritional benefit of YCF consumption. This conclusion is similar to a recent study conducted in the Philippines, where Filipino YCF consumers were reported to have higher likelihood of achieving adequacy in iron, zinc, thiamin, niacin, folate, vitamin B6, vitamin B12, vitamin C, and vitamin D when compared to other milk consumers [20].

The Chinese Dietary Guidelines published in 2016 provide dietary intake recommendations for each food group, including dairy products, which are important food sources for dietary calcium, vitamin D, potassium, protein, zinc, and other nutrients [19]. It is recommended to consume 300 g/day fluid milk or equivalent dairy products on a daily basis. Although dairy products are the main food sources for certain nutrients in Chinese toddlers [6], we found in this study that less than half of the children met the dairy intake recommendation. Previous reports showed the existence of suboptimal nutrient intakes in young Chinese children [21,22,23]. Therefore, increased consumption of dairy products in Chinese toddlers could be an option to provide nutrients commonly lacking in children’s diets. Studies have been conducted to compare the nutrient intakes for consumers of YCF and cow’s milk. A modelling study in Ireland showed that replacing cow’s milk with YCF could increase mean intakes of iron and vitamin D in children 1–4 years [24]. A clinical trial in Germany showed that the consumption of fortified powdered milk with vitamin D improved serum 25-hydroxyvitamin D status in children aged 2–6 years [25]. However, very limited information can be found in China on this topic.

To address this issue, we evaluated two scenarios involving adding the necessary amount of either cow’s milk (model 1) or YCF (model 2) to the diets of children who did not meet dairy intake recommendations. Two hypotheses were tested: first, achieving the recommended dairy intake recommendation will improve nutrient intake; and second, adding YCF and cow’s milk will result in different improvements in nutrient intakes due to the differences in the compositions. A new YCF, which was designed based on the nutritional needs of Chinese children, was used as an example in the analysis. We confirmed that achieving dairy product intake recommendations significantly improved the intake of nutrients. However, increasing cow’s milk or YCF intakes would increase energy intakes by 60–90 kcal. This is still within the recommended energy intake range, however more physical activity or dietary compensation could be needed to maintain energy levels. For the second hypothesis, we found that compared to cow’s milk addition, YCF addition provided lower protein, total fat, and saturated fat intakes. There are studies showing that high protein intake in toddlers is associated with increased BMI and body fat disposition, increasing the risk of overweight and obesity later in life [26,27]. Therefore, lower protein in YCF as compared to cow’s milk is recommended by an expert panel [28]. The total fat intake in the YCF addition scenario was only 1% lower, but the saturated fat intake was 8% lower than the cow’s milk addition scenario, suggesting that the quality of fatty acids is better in the YCF addition scenario. More detailed lipid profile analysis is needed in future research. Regarding the micronutrient intake aspect, cow’s milk provided more riboflavin, calcium, phosphorus, and magnesium, however YCF further improved the intakes of iron, zinc, selenium, potassium, vitamin A, vitamin C, vitamin D, and vitamin B. YCF is more commonly consumed than cow’s milk in Chinese toddlers aged 1–3 years [5]; therefore, adding YCF would be more acceptable and effective than cow’s milk in terms of improving nutrient intake.

The limitations of the present study should be considered when interpreting the results. First, the MING study is a cross-sectional study, so no causal relationships can be concluded. Second, nutrient intakes in the present study were estimated based on a single 24-h recall, so the prevalence of inadequate intake cannot be accurately calculated. Third, Japanese food composition was used to complement Chinese food composition databases in the estimation of vitamin B6, vitamin B12, and vitamin D intakes. Although a group of experts matched the foods carefully, potential inaccuracy needs to be acknowledged. Additionally, data on the content of detailed amino acids and fatty acids were also limited in the Chinese food composition tables, meaning future research is needed to fill the gaps. Fourth, when comparing the nutrient intakes of YCF consumers and non-consumers, we did not further separate children based on their cow’s milk consumption. Since there could be more children consuming cow’s milk in the YCF non-consumer group, the difference between YCF consumers and non-consumers could be bigger than the results showed in the present study.

## 5. Conclusions

In this study, we reported that the percentage of YCF consumption in Chinese children aged 1–3 years is high, especially among children from higher-income families and mothers with higher education levels. YCF contributes significantly to the nutrient intakes of its consumers, who have higher nutrient intakes than non-consumers for iron, calcium, vitamin C, vitamin D, vitamin E, vitamin B6, and folate, all of which are important for growth and immune function. We proved our hypotheses to be true, i.e., diet quality can be improved by achieving dairy intake recommendations and YCF consumption can improve the intakes of minerals and vitamins, while providing a healthier fatty acid profile.

## Figures and Tables

**Table 1:** Percent of children consuming young children formula (YCF) in the study population.

		12–23 Months (N = 470)		24–36 Months (N = 440)		12–36 Month (N = 910)	
		Number of YCF Consumers	% Consuming	Number of YCF Consumers	% Consuming	Number of YCF Consumers	% Consuming
Gender	Boys	262	72.1	225	55.6	487	64.5
	Girls	208	70.7	215	56.7	423	63.6
Family income	Low	137	65.7	110	60.0	247	63.2
	Middle	175	70.9	149	50.3	324	61.4
	High	143	77.6 ^a^	157	58.0	300	67.3
Maternal education	≤9 years	98	53.1	84	42.9	182	48.4
	10–12 years	91	76.9	98	54.1	189	65.1
	≥12 years	266	79.3 ^b^	234	61.5 ^b^	500	71.0 ^b^

Note: ^a^,^b^ *P* < 0.05 when compared with the percentage of children consuming YCF by family income and maternal education, respectively. Chi-square test was used.

**Table 2:** Comparing the daily energy and nutrient intakes between young children formula (YCF) consumers and non-consumers in children aged 12 to 36 months (total N = 910).

Nutrients	YCF	Mean	SD	P25	P50	P75
Energy (kcal)	Non-consumers	978	425	679	911	1267
	Consumers	967	366	689	911	1190
Carbohydrate (g)	Non-consumers	137.5	70.4	86.4	123.5	188.7
	Consumers	131.7	59.0	86.8	121.3	165.3
Protein (g)	Non-consumers	36.4	19.3	22.2	31.6	46.3
	Consumers	34.9	15.4	23.6	32.5	42.9
Fat (g)	Non-consumers	33.6	20.8	19.3	30.1	41.8
	Consumers	34.2	17.2	21.5	31.5	43.7
Saturated fat (g)	Non-consumers	10.5	7.0	5.9	9.3	13.3
	Consumers ^a,^*	6.1	4.7	2.7	5.2	8.5
Dietary fiber (g)	Non-consumers	4.9	5.1	2.0	3.2	6.0
	Consumers	4.1	3.2	2.1	3.3	5.1
Vitamin A (μg RE)	Non-consumers	456.6	449.2	184.0	317.8	598.9
	Consumers ^a,^*	730.2	537.0	371.4	625.1	941.9
Thiamin (mg)	Non-consumers	0.5	0.5	0.3	0.5	0.6
	Consumersa *	0.7	0.5	0.4	0.6	0.8
Riboflavin (mg)	Non-consumers	0.7	0.6	0.4	0.6	0.9
	Consumersa *	0.9	0.7	0.6	0.8	1.1
Niacin (mg)	Non-consumers	6.9	5.4	3.6	5.3	9.3
	Consumers	6.2	3.9	3.6	5.5	8.1
Vitamin B6 (mg)	Non-consumers	0.5	0.4	0.3	0.4	0.6
	Consumers ^a,^*	0.6	0.3	0.4	0.5	0.7
Folate (μg)	Non-consumers	139.9	176.6	60.6	92.6	147.4
	Consumers ^a,^*	145.7	109.0	90.5	126.1	169.6
Vitamin B12 (μg)	Non-consumers	4.7	11.1	0.7	1.4	2.7
	Consumers ^a,^*	3.3	5.0	1.4	2.1	3.4
Vitamin C (mg)	Non-consumers	46.5	211.6	13.1	27.0	43.1
	Consumers ^a,^*	65.2	45.2	36.5	55.4	79.0
Vitamin D (μg)	Non-consumers	11.3	28.0	0.4	1.0	8.3
	Consumers ^a,^*	17.2	35.3	3.5	7.1	20.3
Vitamin E (α-TE, mg)	Non-consumers	9.4	7.8	5.0	8.1	11.7
	Consumers ^a,^*	12.0	6.7	7.0	10.4	15.6
Calcium (mg)	Non-consumers	406.3	285.0	216.9	350.6	536.2
	Consumers ^a,^*	583.8	289.4	379.3	526.2	738.3
Phosphorus (mg)	Non-consumers	615.3	399.9	339.9	544.2	771.2
	Consumers ^a,^	612.2	255.2	431.5	571.8	755.1
Magnesium (mg)	Non-consumers	146.8	80.6	85.7	131.4	197.9
	Consumers ^a,^*	123.7	58.4	82.9	113.0	153.6
Iron (mg)	Non-consumers	11.5	11.3	5.7	8.5	12.8
	Consumers ^a,^*	11.5	6.3	7.9	10.3	13.7
Zinc (mg)	Non-consumers	6.1	3.4	3.5	5.3	8.1
	Consumers ^a,^*	6.4	2.8	4.5	5.9	7.8
Selenium (μg)	Non-consumers	24.5	13.6	14.1	22.4	32.5
	Consumers	23.6	14.1	14.9	21.5	29.8
Sodium (mg)	Non-consumers	2360	1754	1052	2085	3291
	Consumers	2195	1650	879	1871	3379
Potassium (mg)	Non-consumers	929	609	487	790	1173
	Consumers ^a,^*	1021	434	712	952	1251

Note: *P < 0.05 by Wilcoxon signed-rank test, with Bonferroni correction applied for multiple comparisons. ^a^ *P* < 0.05 by multivariate linear regression after log transformation and adjustments for age (months), gender (male or female), income (low, middle, or high), and energy intake. SD, standard deviation; P25, 25th percentile; P50, 50th percentile; P75, 75th percentile; RE, retinol equivalent; α-TE, α-tocopherol equivalent.

**Table 3:** Comparing reported daily energy and nutrient intakes with the addition of cow’s milk (model 1) and a new young children formula (model 2) in children aged 12–36 months who did not meet the dairy intake recommendations (N = 424).

Nutrients		Dietary Nutrient Intake				
		Mean	SD	P25	P50	P75
Energy (kcal)	reported	912	379	631	867	1122
	model1 ^a^	978	376	699	945	1184
	model2 ^a,b^	998	376	720	968	1202
Carbohydrate (g)	reported	128.4	64.3	79.4	115.3	163.3
	model1 ^a^	132.5	64.1	83.3	120.0	167.3
	model2 ^a,b^	138.8	64.2	87.2	125.8	172.7
Protein (g)	reported	33.8	17.2	21.6	30.3	40.7
	model1 ^a^	37.4	17.1	25.6	34.0	44.7
	model2 ^a,b^	36.5	17.0	24.7	32.8	43.7
Fat (g)	reported	30.9	17.0	19.1	28.0	38.3
	model1 ^a^	34.8	17.0	22.6	32.3	41.8
	model2 ^a,b^	34.4	16.9	22.4	31.9	41.2
Saturated fat (g)	reported	7.3	5.4	3.4	6.2	9.6
	model1 ^a^	9.2	5.5	5.6	8.3	11.7
	model2 ^a,b^	8.5	5.4	4.8	7.5	10.7
Dietary fiber (g)	reported	4.6	4.5	2.1	3.2	5.4
	model1	4.6	4.5	2.1	3.2	5.4
	model2 ^a,b^	5.4	4.5	2.9	4.2	6.2
Vitamin A (µg RE)	reported	587.4	530.8	274.1	444.0	775.8
	model1 ^a^	616.6	528.5	304.3	464.9	801.2
	model2 ^a,b^	674.6	525.4	367.1	522.3	861.6
Thiamin (mg)	reported	0.54	0.47	0.34	0.47	0.62
	model1 ^a^	0.58	0.47	0.38	0.50	0.65
	model2 ^a,b^	0.61	0.47	0.42	0.53	0.69
Riboflavin (mg)	reported	0.71	0.51	0.47	0.62	0.79
	model1 ^a^	0.88	0.50	0.65	0.79	0.96
	model2 ^a,b^	0.81	0.50	0.59	0.73	0.89
Niacin (mg)	reported	6.8	5.1	3.6	5.6	8.6
	model1 ^a^	6.8	5.1	3.7	5.8	8.7
	model2 ^a,b^	7.4	5.1	4.3	6.3	9.3
Vitamin B6 (mg)	reported	0.52	0.33	0.32	0.44	0.64
	model1 ^a^	0.56	0.33	0.36	0.47	0.67
	model2 ^a,b^	0.61	0.33	0.41	0.54	0.72
Folate (µg)	reported	149.0	166.9	74.2	110.9	155.5
	model1 ^a^	152.6	166.8	78.6	113.9	158.6
	model2 ^a,b^	169.1	166.4	99.1	129.3	174.5
Vitamin B12 (µg)	reported	4.2	9.8	1.0	1.7	2.8
	model1 ^a^	4.4	9.8	1.2	1.8	3.0
	model2 ^a,b^	4.5	9.8	1.3	1.9	3.1
Vitamin C (mg)	reported	55.4	185.6	23.6	36.8	58.6
	model1 ^a^	56.6	185.6	24.7	38.0	59.3
	model2 ^a,b^	68.4	185.9	36.3	50.5	70.3
Vitamin D (µg)	reported	14.9	39.6	1.6	3.4	15.2
	model1 ^a^	15.2	39.6	2.0	3.6	15.4
	model2 ^a,b^	16.9	39.6	4.2	5.1	17.0
Vitamin E (α-TE, mg)	reported	10.2	6.9	1.9	3.0	4.9
	model1 ^a^	10.5	6.9	6.0	8.9	13.1
	model2 ^a,b^	10.9	6.9	6.4	9.3	13.5
Calcium (mg)	reported	405.4	184.5	274.7	376.1	488.1
	model1 ^a^	532.2	169.6	412.0	492.4	592.5
	model2 ^a,b^	513.4	169.9	394.1	472.2	576.7
Phosphorus (mg)	reported	542.1	244.7	370.7	508.8	653.0
	model1 ^a^	631.1	240.1	462.6	587.6	742.1
	model2 ^a,b^	607.9	240.2	439.1	567.2	711.8
Magnesium (mg)	reported	126.5	68.8	78.4	111.5	161.3
	model1 ^a^	139.9	69.2	92.5	125.8	171.4
	model2 ^a,b^	134.8	68.9	87.4	119.4	167.6
Iron (mg)	reported	11.4	9.6	6.6	9.2	12.6
	model1 ^a^	11.8	9.6	7.0	9.5	13.0
	model2 ^a,b^	12.9	9.6	8.3	10.6	14.2
Zinc (mg)	reported	5.8	2.7	3.8	5.3	7.2
	model1 ^a^	6.31	2.73	4.31	5.79	7.76
	model2 ^a,b^	6.28	2.73	4.29	5.83	7.80
Selenium (µg)	reported	23.9	14.9	14.7	21.5	30.0
	model1 ^a^	26.3	14.8	17.0	24.1	31.9
	model2 ^a,b^	27.2	14.8	17.9	25.0	32.9
Sodium (mg)	reported	2391	1860	970	1974	3414
	model1 ^a^	2436	1859	1028	2036	3451
	model2 ^a,b^	2425	1859	1009	2017	3443
Potassium (mg)	reported	910	501	581	796	1095
	model1 ^a^	1043	496	725	937	1204
	model2 ^a,b^	1061	496	740	955	1220

Note: ^a^ *P* < 0.05 when compared to reported values; ^b^ *P* < 0.05 when compared to models 1 and 2. Paired t test was used in the comparisons. SD, standard deviation; P25, 25th percentile; P50, 50th percentile; P75, 75th percentile; RE, retinol equivalent; α-TE, α-tocopherol equivalent.
